# The entangled Indigenous, rural, and urban realities in Amazônia’s governance

**DOI:** 10.1007/s13280-025-02183-z

**Published:** 2025-04-30

**Authors:** Eduardo S. Brondizio

**Affiliations:** 1 Department of Anthropology, Indiana University, Student Building 236, 701 E. Kirkwood Avenue, Bloomington, IN 47405-7100 USA; 2 Environment and Society Program, University of Campinas, Cidade Universitária Zeferino Vaz - Barão Geraldo, Campinas, SP 13083-970 Brazil

**Keywords:** Amazônia, Climate change, Environmental governance, Illegal economies, Indigenous people, Sociobioeconomy

## Abstract

As Amazônia takes center stage at the UNFCCC COP30 climate summit in November 2025, attention has justifiably turned to the urgency of preventing tipping points in its forest-climate balance. Underlying this scenario is a crisis of environmental degradation, social inequalities, urban precarity, and violence; these intertwined realities, often hidden by simplistic imaginaries, are inseparable from the climate crises. The bold vision that previously established an ambitious system of territorial rights and environmental governance should now inspire strategies to confront the pressures that are eroding these advances. A new social contract must reckon with the complexity of interconnected crises driving the region toward tipping points. These strategies must safeguard Indigenous and traditional communities’ territories while extending environmental governance to urban, peri-urban, and rural areas. Building social capital for collective action among conflicting actors is the region's most significant challenge; polycentric governance approaches can bridge arrangements to ensure the basin’s health.

## Confronting simplistic and enduring Amazonian imaginaries

In November 2025, the UNFCCC climate summit—the 30th Conference of the Parties (COP30)—will place Amazônia once again at the forefront of the global stage at this pivotal moment in history. But just like other regions in the Global South already hit hard by climate change, Amazônia cannot wait for a fragmented international community to resolve its collective action challenges. Avoiding an impending biophysical tipping point for Amazônia depends on addressing climate change globally while tackling environmental degradation regionally (Flores et al. 2023).

A central question on the global front is whether COP30 can break the pattern of past summit failures and secure meaningful commitments to reduce emissions—especially in an era of social media-driven disinformation, political polarization, renewed commitment to fossil fuel, and stronger autocracy worldwide. The question on the regional front is whether responses to revert a biophysical tipping point will be pursued in isolation or integrated with solutions for the interconnected crises of environmental degradation, social inequalities, and violence spreading across the region. Nowhere are these crises more deeply intertwined than in the Brazilian Amazon^1^. Yet, they are often obscured by popular imaginaries, rooted in colonial legacies that depict the region either as an untouched wilderness or a mere resource frontier. All those engaged with the future of Amazonia—including policymakers, researchers, civil society, and the private sector—must reckon with the full complexity of its interconnected crises—realities that Indigenous, rural, and urban Amazonians navigate daily.

The enduring images of Amazônia as a pristine wilderness and/or as a land of self-sustaining Indigenous communities contrast sharply with its current struggles. Romanticized visions of pristine wilderness and untouched forests ignore both the millennia of Indigenous’ forest and agriculture management and domestication (Levis et al. 2017) and the profound and extensive human impacts on its ecosystems today (Lapola et al. 2023). Similarly, imaginaries that isolate Indigenous and local communities from broader socioeconomic and political contexts obscure the complexity of cultural, health, and territorial threats communities navigate daily, as well as the multiple environmental and economic contributions they provide within and beyond their territories. At the same time, agro-technotopian imaginaries that portray Amazônia as a potential industrial monocrop and mining landscape are not just misguided—they are dangerously irresponsible. They perpetuate the same extractive logic that has historically driven cycles of dispossession and environmental degradation in the region. These imaginaries come with—legally and illegally—financed efforts that are destroying forests, rivers, and cultures while deepening social inequities. And while urban areas remain absent from all imaginaries of Amazônia, the cities where 80% of the population live, including around 40% of the regional Indigenous population, are now central to the region’s present and future—and arguably represent its greatest predicament.

A significant portion of Amazônia today is as urban as it is Indigenous and rural. All three realities are already deeply intertwined, shaping and reshaping one another and defying simplistic categorization or solutions. Drought and flood extremes, deforestation and fires, ubiquitous pollution from mining, pesticides, smoke, and sewage spread to and from rural and urban areas. Protected territories are progressively becoming islands of biological and cultural diversity slowly eroded by legal and illegal economies underlying deforestation and land occupation, mining, logging, and pollution (RAISG 2020). Organized crime and illegal economies are thriving within this vulnerable and fragmented landscape (Igarapé Institute 2022, 2024; UNODC 2023; FBSP 2024). These social realities can no longer be disentangled from the environmental and climate crises that have long dominated public discourse. Amazônia is not a singular ecological entity but a complex socioenvironmental system whose future depends on recognizing these entanglements and the systemic risks they pose—and reimagining the region’s governance accordingly. The functional interdependence of Indigenous, rural, and urban areas means that environmental governance must safeguard Indigenous territories, sustainable use and protected areas but also transcend them to engage with the multiple, diverse, and interconnected realities of all Amazônia (Hecht et al. 2024). This is a shared responsibility not only among all Amazonians, but also of non-regional actors whose decisions, investments, and policies influence changes in the region.

The Amazônia we discuss today cannot be separated from its historical context. The region’s social conditions and urgent needs are obscured by enduring imaginaries of Amazônia, shaped by colonial legacies and the persisting prevalence of external interests over regional demands. The region is often treated as an object of national and global priorities rather than as a place with its own histories, local governance systems, needs, and solutions for the future. These imaginaries do not accommodate the messiness of urban areas and illegal economies, nor the successes and needs of Indigenous and local communities to confront regional problems. As the international community prepares for the historic COP30 in Belem, efforts to prevent Amazônia’s biophysical tipping point must go hand in hand with addressing and dismantling these simplistic and enduring imaginaries to ensure that Indigenous, rural, and urban communities are empowered in environmental governance and climate action.

## The impacts of climate change are accelerating and amplifying in Amazônia

There is growing evidence that Amazônia is moving toward subregional biophysical tipping points, with expected forest diebacks and changes in terrestrial and aquatic ecosystems composition, structure, and functioning (Flores et al. 2024). Focusing on addressing these concerns is justifiable, and the region’s unique biological and ecosystem diversity plays a critical role in global and continental climate regulation and freshwater cycles (Boulton et al. 2022). Furthermore, Indigenous communities and marginalized rural and urban Amazonians are experiencing the most severe impacts of climate change (Bowman et al. 2021). This was particularly visible during the record droughts and burning seasons over the past two years (Santos de Lima et al. 2024) and previous years of record flooding.

A recent report from the NGO InfoAmazonia estimated that more than half of the municipalities in the Brazilian Amazon experienced drought throughout the entirety of 2024 (InfoAmazonia 2025). The dry period across the southern portion of the region now lasts over 3–5 months (Costa et al. 2024a). Extended dry conditions and more flammable landscapes significantly increase the risk of accidental fire on already economically vulnerable and poorly capitalized communities (Brondizio and Moran 2008; Cammelli et al. 2020). Extreme droughts and accidental fires are directly impacting local management systems in forestry and agroforestry. Producers have limited access to extension services and credit to implement irrigation systems or other protections. The same droughts together with changing flooding cycles are altering the management cycles of fisheries, by evaporating entire rivers, changing water temperature beyond threshold levels for fish and mammal species, and impeding avenues of transportation essential for people to reach needed social services and to get perishable and precious fish, fruit, produce, tubers, seeds, and oils to the market (Santos de Lima et al 2024). Urban areas are also suffering from the effects of climate extremes such as drought, floods, and extreme heat, and which are compounded by infrastructural precarity and pollution from sewage, mercury, pesticides, and particulates from burning. The region urgently needs new environmental and climate governance approaches that recognize the growing functional interdependencies of Indigenous, rural, and urban landscapes. Although these interdependencies intensify climate change impacts on the one hand, they also create opportunities for amplifying and strengthening local responses by connecting initiatives across these landscapes.

## The imperative of preserving advances in environmental governance and territorial rights

Arguably, the most important actions taken to protect Amazônia and its people during the past three decades have been the recognition and demarcation of territorial rights for Indigenous people and traditional communities, and the implementation of a network of protected and sustainable use areas across the region (RAISG 2023; Josse et al. 2024). The recognition of territorial rights has been pivotal for reducing deforestation rates, promoting ecosystem restoration, protecting waterways, conserving biodiversity and agrobiodiversity, preventing GHG emissions, and promoting carbon sequestration (Baragwanath and Bayi 2020; Quintanilla et al. 2022). However, these advances are being eroded and undermined by a combination of legal efforts by agribusiness and mining lobbies, such as in the Brazilian national congress, and by illegal, violent threats on the region.

Indigenous and protected areas cover about 50% of the basin and nearly 45% of the Brazilian Amazon. They have been cradles of innovation and advancement in economies that are grounded in the sustainable use and management of forest and river resources (Brondizio et al. 2021; Londres et al. 2023). These areas have become incubators of innovation in agroforestry, community-based management of fisheries and forests, restoration techniques, and initiatives creating value-aggregation industries for local products through producer’s cooperatives and associations (Garrett et al. 2024). Today, dozens of products derived from native fruits, medicinal plants, seeds and oils, and fisheries are reaching regional, national, and international markets, employing hundreds of thousands of people in the region, and central to the economic life of municipalities (Brondizio et al. 2021; Costa et al. 2022). These locally led efforts, or *sociobioeconomies* (i.e., biodiversity-based production systems rooted in knowledge and management practices of Indigenous and rural communities), have brought Amazonian *sociobiodiversity* to the world (i.e., a wide range of resources, products, and managed landscapes resulting from the region’s biological diversity along with knowledge and practices of Indigenous and local communities) (De Castro et al. 2024). They have demonstrated the potential for an Amazonian economy that is more inclusive, environmentally sustainable, generating employment, and aligned with territorial governance (Campos-Silva et al. 2021).

Sociobioeconomies transcend protected territories and play a major role in regional conservation and the larger regional economy. For instance, from the hands of riverine families the açaí fruit (*Euterpe oleracea*) economy has expanded to become the region’s most important activity in terms of the number of municipalities participating, economic scale, and employment in rural, urban, and Indigenous areas (Brondizio 2008, 2020). The revolution in the region’s cacao production is equally notable. Rural and Indigenous producers in different parts of the region are delivering world class cocoa beans and producing their own chocolate, having previously only exported low graded beans. In another remarkable example, community-based management (CBM) of fisheries led to the recovery of Amazônia’s most emblematic and endangered fish, the giant Pirarucu (*Arapaima gigas*) in a span of only 20 years. As a result, riverine communities have been granted governance rights over floodplain lakes, which has improved food security, income, and access to basic services (Campos-Silva et al. 2021; Franco et al. 2021). These communities contribute to the direct and indirect protection of vast areas of forests, rivers, and lakes, providing socioeconomic and environmental benefits of regional significance (Rodrigues et al. 2025). Likewise, planted agroforestry systems are spreading along rivers and road networks to farmers at all scales, showing its potential for food production, restoration and carbon sequestration, and rural development (Futemma et al. 2020). Examples like these abound under the shadows of the forest.

However, even the most successful sociobioeconomies are facing challenges to maintaining their economic viability, as well as growing pressures from illegal markets and organized crime. Despite their importance, Amazonian sociobioeconomies are statistically invisible because of an enduring imaginary that sees Indigenous and rural sociobioeconomies as localized and irrelevant to the larger regional and national economy. Sophisticated and highly productive agricultural, agroforestry, and forest management systems that do not conform to expectations of monocultural production systems are easily dismissed and labeled as extractivism of native forests or mere subsistence systems (Brondizio 2020). Data on the significance of the production of sociobiodiversity, employment, value-aggregation, and environmental benefits are limited, unreliable or inexistent.^2^ This is particularly true for sociobioeconomies that operate through fragmented and informal transactions. This lack of visibility, when compared to conventional national exports such as soy, beef, and palm oil, translates into a lack of social value and recognition for these producers, limiting their access to credit and investments in logistical infrastructure that is fit for the purpose and scale of diverse sociobioeconomies.

To safeguard the advances of sociobioeconomies and build resilience to climate change, we must challenge two key assumptions upon which environmental governance has been built since the 1990s. The first assumption is that area-based environmental governance can provide both territorial rights and protection against ecosystem degradation. While this assumption has, until recently, served as a [relatively] effective buffer for deforestation, logging, illegal fishing and poaching, and mining, it is increasingly insufficient on its own. Environmental degradation, often driven by external economic and political pressures, continues to encroach upon these territories. The lack of law enforcement, accentuated since the Bolsonaro government (2019–2022), leaves communities as the first line of defense against powerful and violent actors. Furthermore, during the past two decades, inter-urban networks have expanded and connected previous isolated urban areas. This process is consolidating corridors of cleared forests, a vast network of informal roads, extensive pastures and monocultures, mining, and infrastructure. These transformations are not random; they are symptoms reflecting enduring patterns of territorial occupation that have historically prioritized extraction over sustainability. As a result, they are fragmenting and polluting the region’s watersheds haphazardly, encircling and undermining the environmental health of Indigenous territories, traditional communities, and protected areas, impacts that also affect urban communities.

The second assumption relates to an expectation that strong markets for locally managed, sustainable, and biodiverse production systems can provide a pathway for reconciling conservation with local and regional economic development and social inclusion in Amazônia (Brondizio 2011). The first part of this equation has been realized. As mentioned above, Indigenous and rural communities have long adapted and refined a multitude of innovations in agroforestry, forest, and fisheries management (Brondizio et al. 2021). This has opened markets for valuable biodiversity products, used in industries and commercialized around the world today. However, the second part of this equation of maintaining their economic viability remains deeply challenging. Unfair pricing set by intermediaries, precarious or inexistent access to logistical infrastructure, and high costs to monitor and protect territories from illegal activities are eroding the economic viability even for the most valuable products. These challenges and barriers are not merely market inefficiencies, and they reflect structural inequalities that have long burdened those who have historically resisted and creatively sustained their territories and economies. Nowadays, the persistence of these historical inequalities and structural problems are discouraging the youth from continuing to build on these hard-fought achievements.

Initiatives addressing these shortcomings exist and can be strengthened and built upon (Londres et al. 2023), but a broader framework to support the region’s sociobioeconomies is needed (Marcovitch and Val 2024; Garrett et al. 2024). In some areas, Indigenous groups have reached beyond their territorial borders to promote restoration and connectivity for the health of rivers and forests (Brondizio et al. 2009; Sanches et al. 2021). Community-based cooperatives and value-added industries have been able to develop fairer contracts directly with industries. Institutional support, such as requiring municipalities to buy a portion of school lunches from smallholder producers, has proven key to local rural development and could be scaled in scope and to other sectors, like military bases and hospitals. New forms of network-based governance involving community organizations, NGOs, and local and regional authorities have also emerged, such as the Pirarucu Collective, the Xingu Seeds Network, Origens Brasil, and the Brazil Nut Collective, among others (De Castro et al. 2024), to address environmental governance challenges that transcend territories. They are also improving access to market logistics and price negotiation and exploring new ways to bring payment for environmental services and/or carbon schemes to those who are producing sustainably and protecting and caring for forests and rivers. These existing and emerging efforts need to be recognized and supported as key to climate action in the region.

## Environmental governance of Amazônia must include urban areas

In less than 50 years, through phases of state-driven infrastructure and settlements, frontier expansion, and internal migration, the Brazilian Amazon has seen the emergence of hundreds of new municipalities and, consequently, urban centers (Amazônia 2030). Today, the Brazilian Amazon includes over 770 municipalities, the majority of which were formed after 1980. Over 60% of municipalities have populations smaller than 20 000, and over 95% of them are smaller than 100 000. The 2022 Brazilian census shows over 22 million people live in urban areas of the Brazilian Amazon, including over 375 000 Indigenous or near 40% of the regional Indigenous population. Despite their demographic, political, and economic importance, urban areas remain largely neglected and virtually invisible in most discussions about regional development and environmental problems (Brondizio 2016). At the same time, urban centers have become crucial to any solution envisioned for Amazônia—whether in governance models, value-added processing industries of sociobiodiversity products, infrastructure development, enforcement of environmental laws, and/or training and capacity-building programs.

Amazonian municipalities are among the most precarious in services and economically insolvent in Brazil (Hanusch 2023). Urban precarity and subnormal occupation^3^ are the norm for cities in Amazônia. Half of the 20 most populous subnormal occupations in Brazil are in Amazônia. This is the predominant reality in Amazônia’s largest cities such as Belem, with 57%, and Manaus, with 56%, of their residents in subnormal occupations (IBGE 2024), and a reality that is present in cities of all sizes. Precarity in cities drives illegal economies in rural and Indigenous areas, and vice versa. Organized crime has increased 46% from 2023 to 2024, with 19 organizations spreading to one-third (or 260) of Amazonian municipalities in Brazil (FBSP 2024). It has exacerbated the region’s homicide rate, which is 40% above the Brazilian average. Its tentacles expand far into the region’s floodplains, deep forests, and Indigenous lands and involve illegal mining, fishing, logging, deforestation, and land grabbing (Costa et al. 2024b; Igarapé Institute 2024). These illegal economies and the illegal occupation of public and Indigenous lands are intertwined with legal agricultural and natural resource value chains (Igarapé Institute 2022; Costa et al. 2024b) and, in some cases, with national and international drug trafficking (UNODC 2023). Organized crime is becoming a major ‘employer’ in the region, co-opting or forcing the youth in urban, rural, and Indigenous areas into drug trafficking, environmental crimes, prostitution, and human trafficking.

There are strong social connections, high levels of mobility, and economic interactions among urban, rural, and Indigenous areas (Eloy et al. 2014; Padoch et al. 2007). Indigenous urban residents, even more invisible in this context, maintain strong connections to their territories. Rural and Indigenous sociobioeconomies are the backbone of municipal and urban economies, circulating money in local commerce, providing the bulk of fresh food and regional staples such as manioc flour, fish, açaí, and other fruits, and a multitude of resources for urban markets. While rural areas provide resources as sources of income, the city provides access to some public services, goods, informal jobs, and commercial opportunities (Brondizio et al. 2013). This close connection between rural and urban areas, along with the depopulation of government-planned agrarian settlements, has contributed to the accelerated urbanization of the region and the resulting demand for public services.

To understand why urban areas are absent from environmental and climate discussions, it is essential to examine two key historical disconnections. The first is between Amazonian municipal economies and the region’s natural resource and agricultural economies, which helps to explain the insolvency of municipalities and their incapacity to confront the precarity of urban areas (Brondizio 2011). Sociobiodiversity and mining value chains worth billions of dollars flow across Amazonian municipalities mostly through informal networks of workers and producers, intermediaries, local businesses and consumers, and corporate buyers. The same informality exists, at least in part, in the cattle ranching and agricultural economy. Municipalities are largely unable to capture taxes from these economies because of a lack of infrastructure for commercialization and value-added industries, which make them dependent on transfers from the federal and state government. Most formal jobs are in the municipal public sector, while most working-age people have informal occupations. Most municipalities face cumulative deficits between service demand and provisioning and are, thus, unable to meet the accelerating demands for public services such as for education, health, sanitation, and safety, both in urban and rural and Indigenous areas within their borders (Hanusch 2023). Such conditions have become fertile ground for illegality, which extends from urban centers to the most distant corners of the Amazon (Amazon Underworld 2023). In fact, illegal economies operate openly in many urban markets (e.g., fish, wildlife, timber, gold), which is indicative of the level of pressure and lack of enforcement in Indigenous and rural areas.

The second disconnection happens between urban elites, which often control municipal administrations, and socioenvironmental discussions in Amazonia. This disconnection is not merely incidental but historically entrenched. Urban political elites often originated from families whose economic foundations were or are built on extractive industries—logging, mining, and large-scale agriculture and ranching—practices that prioritized short-term profit, large-scale land occupation, and labor exploitation over environmental sustainability and social concerns. This legacy continues to shape governance, as many of these families remain embedded in an extractive economic logic, reinforcing policies that favor resource exploitation and wealth concentration rather than sustainable development. Consequently, socioenvironmental concerns struggle to gain political traction, thereby marginalizing efforts toward conservation and equitable resource management. In fact, this disconnection has become further entrenched as a reflection of national political trends. For instance, the 2022 election results show a strong correlation between voting for the far-right promoting anti-environmental narratives and deforestation trends during the last four decades. These are also municipalities with a prevalence of public lands grabbing, illegal mining and logging in Indigenous territories and protected areas, expulsion of traditional communities, environmental degradation practices, and threats against leaders and environmentalists (Canale et al. 2024). Urban areas are also subjected land grabbing and illegal occupation by organized crime, a process which is also connected to illegal forest logging and timber markets. 

Urban residents are also suffering from impacts originating in distant regions. Illegal mining, indiscriminate use of agrochemicals, and fire affect urban residents with pesticides and mercury pollution in their water and food, and high levels of air pollution from burnings (ex. Meneses et al. 2022). The degradation of rivers and waterways exacerbates the vulnerability of urban residents who tend to live in low-lying areas in precarious residences with limited access to basic infrastructure (Dias et al. 2021). Only a fraction of Amazonian residences has a connection to sewage systems in small pockets of urban areas, of which only a fraction receives any treatment. Lack of sanitation compounds the health impacts of flooding and the scarcity of clean water during droughts and spreads pollution to surrounding riverine areas (Mansur et al. 2016).

Given the scale of precarity, urban areas need investments in climate adaptation measures, including nature-based solutions, to increase the resilience of urban communities to drought and flood extremes and decrease the impact of urban pollution on surrounding ecosystems (Schaeffer et al. 2023). While adaptation measures and infrastructure represent massive undertakings in the region’s few large cities, these could represent pathways for transformative change in the many small and mid-size cities of the region, before such problems become unmanageable.

## Toward environmental governance that reconnects Amazonian imaginaries with reality

The future of Indigenous, rural, and urban communities in Amazônia has always been intertwined, yet governance frameworks have historically treated them as distinct domains of policy and research. This myopic view is no longer an option. The bold vision that, three decades ago, established one of the most ambitious systems of territorial protection, social inclusion, and environmental governance in Amazônia should now inspire an equally ambitious regional strategy to confront its complex governance challenges. This strategy must safeguard the rights and territories of Amazonian Indigenous people and traditional communities while extending environmental governance to incorporate urban areas and their surrounding peri-urban and rural landscapes. For environmental governance to be effective, it must also confront the region’s colonial and neocolonial legacies that continue to shape development narratives, access to land, public investments, credits and subsidies, and decision-making.

Increasing misfit between existing governance arrangements and environmental change calls for a bold and renewed vision for environmental governance that is polycentric in nature, breaking from models that, for too long, have privileged external interests and decisions over local realities and voices. A polycentric approach aims at coordinating efforts at the regional level to guarantee the environmental health and integrity of the basin with supporting overlapping but independent governance arrangements at meso- and local levels. A polycentric governance approach can contribute to foster the development of bridging institutions to facilitate environmental governance, for instance, of watersheds, or other units, that transcends Indigenous and protected territories and permeate rural and urban landscapes. Recognizing and strengthening connections between meso-level (e.g., watershed) and local governance systems (e.g., territories; rural properties and settlements, networks of actors and community organizations) is crucial for reflecting the region’s diverse social contexts and forms of collective action, environmental conditions, and for encouraging bottom-up initiatives mobilizing knowledge to respond to context specific challenges. As mentioned above, examples of network-based governance involving diverse actors across large regions exist and can serve as inspirations. The complexity, nonlinearity, and scale of regional problems may give the sense that the impact of local-level actions and initiatives will be dampened at larger levels. Conversely, it also opens the possibility that these actions have the potential to be amplified to drive significant impacts at larger levels (Brondizio et al. 2016).

How new models of governance are implemented matters. The last fifty years of governance interventions in the region testify that externally imposed ideas, projects, and investments are doomed to fail (Londres et al. 2023). Even though polycentric governance foregrounds principles of recognition and procedural justice, any governance intervention must be designed by the actors who will play a central role in the governance arrangement. This ensures that relevant stakeholders are involved and that priorities align with the spatial–temporal contexts and dynamics of territories and local ability to maintain, adapt, and advance governance arrangements in the face of climate change and other pressures. Any governance structure implemented in a top-down manner will lack the legitimacy necessary for its proper functioning, even if its goal is to be participatory.

In this context, the most difficult challenge facing the region is building the needed social capital for collective action among contrasting and politically conflicting actors, with different roots and connections to the region, and visions for its future (Brondizio et al 2009). No financial commitment achieved at COP30 can solve this challenge. Building a new social contract will require not only connecting contrasting Amazonian imaginaries but also reckoning with the structures of inequality—historically entrenched in governance, land tenure, and resource distribution—that continue to push the region’s trajectories to a biophysical tipping point.

Amid current political schisms, it would be a naïve panacea to think that COP30 will represent a turning point for the predicaments that face the region and the world. Beyond the expected pragmatic measures, COP30 should serve as a catalyst for a new paradigm for environmental governance in Amazônia, which addresses not just the urgency of climate action but also the long-standing asymmetries of power and responsibilities that have shaped Amazônia’s crises. There is only so much that Indigenous and local communities, NGOs, social movements, or  responsible farmers and market schemes can shoulder to confront the Amazonian crises. The broader challenge is to ensure a new development vision of Amazônia that is shared among diverse stakeholders, from local to international levels. Environmental governance for all also demands responsibility by all. A sense that everyone depends on the health of the basin and the health of the basin depends on everyone.

It is time to cultivate new imaginaries that acknowledge these interdependencies, not as abstract principles, but as guiding frameworks for action— imaginaries that confront historical injustices while elevating the solutions emerging from the ground. It is also time for concrete actions and financing aligned with the needs in climate adaptation, security, and economic opportunities of the most marginalized Amazonians. Addressing the region’s social challenges is not just a moral imperative, it is a necessary step toward dismantling long-standing inequalities that undermine environmental governance and, ultimately, efforts to avert Amazônia’s looming biophysical tipping points and its regional and global consequences.

## Footnotes

1 While this text focuses mostly on the Brazilian Amazon, in many ways, the issues discussed on this side of the border reflect that of countries across the basin.

2 As a positive sign, however, the Brazilian census on agriculture, forest, and aquaculture intends for the first time to include Indigenous and traditional communities as part of its 12th edition (2025–2026).

3 IBGE defines subnormal occupation as informal and irregular occupation of urban land marked by lack of formal recognition or ownership, precarious infrastructure, poor housing conditions and high-density occupancy, and overall conditions of vulnerability and social exclusion.
